# Role and expression of FRS2 and FRS3 in prostate cancer

**DOI:** 10.1186/1471-2407-11-484

**Published:** 2011-11-11

**Authors:** Tania Valencia, Ajay Joseph, Naveen Kachroo, Steve Darby, Susan Meakin, Vincent J Gnanapragasam

**Affiliations:** 1 Translational Prostate Cancer Group, Department of Oncology, Hutchison/MRC research centre, University of Cambridge, Cambridge, UK; 2 Northern Institute for Cancer Research, University of Newcastle, Newcastle upon Tyne NE2 4HH, UK; 3 Laboratory of Neural Signalling, Robarts Research Institute, London, Ontario, Canada

**Keywords:** Prostate cancer, FGF signalling, FRS2, FRS3, Adaptor proteins

## Abstract

**Background:**

FGF receptor substrates (FRS2 and FRS3) are key adaptor proteins that mediate FGF-FGFR signalling in benign as well as malignant tissue. Here we investigated FRS2 and FRS3 as a means of disrupting global FGF signalling in prostate cancer.

**Methods:**

FRS2 and FRS3 manipulation was investigated *in vitro *using over-expression, knockdown and functional assays. FRS2 and FRS3 expression was profiled in cell lines and clinical tumors of different grades.

**Results:**

In a panel of cell lines we observed ubiquitous FRS2 and FRS3 transcript and protein expression in both benign and malignant cells. We next tested functional redundancy of FRS2 and FRS3 in prostate cancer cells. In DU145 cells, specific FRS2 suppression inhibited FGF induced signalling. This effect was not apparent in cells stably over-expressing FRS3. Indeed FRS3 over-expression resulted in enhanced proliferation (*p *= 0.005) compared to control cells. Given this functional redundancy, we tested the therapeutic principle of dual targeting of FRS2 and FRS3 in prostate cancer. Co-suppression of FRS2 and FRS3 significantly inhibited ERK activation with a concomitant reduction in cell proliferation (*p *< 0.05), migration and invasion (*p *< 0.05). Synchronous knockdown of FRS2 and FRS3 with exposure to cytotoxic irradiation resulted in a significant reduction in prostate cancer cell survival compared to irradiation alone (*p *< 0.05). Importantly, this synergistic effect was not observed in benign cells. Finally, we investigated expression of FRS2 and FRS3 transcript in a cohort of micro-dissected tumors of different grades as well as by immunohistochemistry in clinical biopsies. Here, we did not observe any difference in expression between benign and malignant biopsies.

**Conclusions:**

These results suggest functional overlap of FRS2 and FRS3 in mediating mitogenic FGF signalling in the prostate. FRS2 and FRS3 are not over-expressed in tumours but targeted dual inhibition may selectively adversely affect malignant but not benign prostate cells.

## Background

The Fibroblast Growth Factor (FGF) pathway is an important stimulus to cancer development and progression in many epithelial tumours [1-3]. In prostate cancer many FGF ligands and FGF receptors (FGFR) have been shown to be significantly over-expressed [4-8]. Targeted inhibition of FGF signalling has shown promise in *in vitro *studies but has so far not translated into clinically useful therapies. A key problem is the inherent functional redundancy in a system that has 23 known ligands and 4 receptors [1,2]. All FGFs however require the FGF receptor substrate (FRS) 2 and 3 adaptor proteins to initiate down-stream signalling [9]. These proteins act as coning centres and are crucial for recruitment and activation of the MAPK and other signalling cascades following phosphorylation of FGFRs.

FRS2 and FRS3 share 49% sequence identity and contain a N-terminal myristylation domain important for membrane anchoring, a phospho-tyrosine binding (PTB) domain through which they interact with FGFRs and a C-terminal domain with tyrosine phosphorylation sites for Grb2 and phosphatase Shp2 [9]. The FRS2-Grb2-Sos and FRS2-Grb2-Gab1 complexes activate the RAS/MAPK pathway and the PI3 kinase pathway, respectively [10]. FRS2 not only binds to FGFRs, but also to the TrkA, VEGF and RET receptors suggesting a broad role in signal transduction [11,12]. FRS2 plays an important role in cell differentiation, proliferation, migration and cycle arrest [13-16]. FRS2 null mouse die at E7.0-E7.5 due to impairment in development [17]. The function of FRS3 is less well understood and FRS3 null mice have not been reported. Ectopic expression of FRS3 in fibroblast derived from FRS2 -/-mouse embryos however has been shown to rescue FGF induced ERK activation suggesting functional overlap in embryogenesis [18]. FRS3 has been proposed to have an important role in negative regulation of EGF receptor signalling [19,20] and recently has been reported as a novel microtubule-associated protein [21].

FRS sits at the critical juncture between the FGFR and downstream signal transduction. It is therefore a potentially attractive target to disrupt the mitogenic and tumourigenic effects of multiple FGFs. An important first step however is to determine the relative expression and function of FRS2 and FRS3 in a specific cancer. There have been very few studies of FRS2 and FRS3 in clinical cancers and no reports in prostate cancer. In lung cancer, reduced FRS3 was associated with a poor clinical prognosis [19]. Conversely, in thyroid cancers FRS3 expression was unchanged but FRS2 expression was increased [22] suggesting potential tissue specific FRS2 and FRS3 changes in tumours. In this study we investigated the relative expression and functional role of FRS2 and FRS3 in prostate cancer.

## Methods

### Cell lines and stable construct

Malignant and benign prostate cell lines were cultured in RPMI (Invitrogen) supplemented with 10% FBS. All cell lines used (LNCaP/DU145/PC3/PNT1A/PNT2) were purchased commercially (American Type Culture Collection or Health Protection Agency Culture Collections). 2 μg of myc-tag pCDNA3.1-FRS3 (received from Dr S Meakin) or pcDNA3.1 empty was transfected using Lipofectamine 2000 (Invitrogen). Transfected cells were placed under G418-sulphate selection for 14-20 days. Individual colonies were removed by trypsinization and expanded, and clones screened for FRS3 expression. For androgen induction experiments, LNCaP cells were initially grown for 24 hours in standard media and then maintained in charcoal stripped serum supplemented media for 24 hours. Following this, synthetic androgen (R1881) was added at a dose of 10 nM. Cells were harvested at the indicated time points and RNA extracted for real time PCR analysis of FRS2 and FRS3 and PSA expression.

### siRNA and Real time polymerase chain reaction

Cells were grown for 24 hours prior to transfection. 33 nM of siRNA oligonucleotides: Silencer^(R) ^Select Pre-designed siRNA FRS2, s21262 (Applied Biosystems) and ON-TARGETplus FRS3, L-019038-00-0020 (Dharmacon) was used to transfect cells. Silencer^(R) ^Select Negative Control #1 siRNA, 4390844 (Applied Biosystems) was used in parallel as the scramble control. The results presented are the mean value of six separate set of experiments. Total RNA was extracted using RNeasy Mini Kit (Qiagen) and reverse transcribed using Transcriptor cDNA Synthesis Kit (Roche Diagnostics) according to the manufacturer's instructions. Real time sequence specific Taqman PCR primers for human FRS2 (Hs00183614_m1), FRS3 (Hs00183610_m1) and GAPDH (Hs02786624_g1) were purchased from Applied Biosystems. qPCR was preformed with Light Cycler 480 (Roche Diagnostics) and Taqman Universal PCR Master Mix (Applied Biosystems) and corrected for GAPDH expression. PCR was repeated in triplicate and performed three times with results expressed as the mean and standard deviations.

### Cell proliferation assays

Cells were seeded at a density of 3000 (DU145, DUEV, DUFRS3) and 5000 (PC3, PNT2) cells in 96-well plates and allowed to grow for 36 h. Medium lacking serum was termed 'basal medium' (BM), while medium containing 10% FBS was termed as "full medium" (FM). Cells were then starved in BM for 16 h before stimulation with FM. Cell proliferation was assessed with WST-1 reagent (Roche Diagnostics). Experiments were repeated in triplicate and done three times.

### Western blotting

Cells were lysed in Laemmli buffer and denatured. Samples were then separated using 10% Bis-Tris pre-cast gels (Invitrogen), followed by transfer to a PDVF membrane (GE Healthcare). Primary antibodies: FRS2-A5 (sc-17841), pERK-E4 (sc-7383), ERK-6G11 (sc-81458), c-Myc-9E10 (sc-40) were purchased from Santa Cruz Biotechnologies, Abcam (α-tubulin, ab4074-100) and BD Biosciences (PARP, 65196E). FRS3 antibody was received from Dr S Meakin and has been previously described [22]. Primary antibody complexes were detected using HRP-conjugated secondary antibodies (Dako). Protein bands were visualised using ECL (GE Healthcare).

### Migration and invasion assays

Transfected cells were re-plated out into BD migration or invasion chambers (Scientific Laboratory Supplies) in basal medium (BM) at a concentration of 80, 000 cells per insert. FM or FGF1 (10 ng ml^-1^), FGF2 (10 ng ml^-1^) and FGF8 (10 ng ml^-1^) prepared in basal media were used in the lower chamber as chemoattractant. Cells were allowed to migrate or invade for 24 hours and fixed in methanol for 20 minutes at -20°C, stained with haematoxylin, washed with dH_2_O and allowed to dry before mounting them in a microscopic slide with DPX (Sigma Aldrich). Cells were counted using a bright field microscope at 20× magnification. Five different fields of view were used to obtain an average count per section. Results shown are the mean of three experiments and expressed as a fold increase over un-induced scramble controls (migration) or as invasion index (compared to and corrected for control inserts). Statistical analysis was performed using two-tailed Student's T-test. *p *< 0.05 being significant.

### Radiation survival assays

Cells were down-regulated for FRS2 and FRS3 expression by siRNA as described and re-plated at 1000 cells per well in 6-well plates and left to grow for 24 h. Cells were then subjected to different doses of radiation (1 Gy and 3 Gy) *in situ *and left for 10 days. At the end of this period dead cells were washed off and residual colonies were Giemmsa stained and counted. Counts were taken in four separate areas and averaged for each plate. Experiments were repeated in triplicate and done three times. Results are expressed as a percentage compared to non-irradiated cells.

### Clinical biopsy laser micro-dissection and sample preparation

Diagnostic prostate biopsies from men with histological proven prostate cancer were identified from a pathology resource and used in accordance with ethical approval gained from Cambridgeshire local ethics committee in 2009 (Ref: 09/H0308/42). All samples were anonymised at source. As all samples were archival, collected surplus to diagnostic need and anonymous at the point of collection no specific patient consent was deemed necessary by the ethics board for use of these samples. Formalin fixed paraffin embedded tissue (FFPE) excess to diagnostic need were sectioned at 10 μm thickness onto Polyethylene napthalate (PEN) membrane slides (Leica TM). Slides were deparaffinised twice with xylene, rehydrated using graduated ethanol/DEPC water then H&E stained. Slides were pre-marked for benign or malignant areas and by Gleason grade (Grades 3/4/5) using the diagnostic matching slide as a guide. Pre-marked slides were then micro-dissected using a Leica TM LMD6000 system. Micro-dissected tissue was then subjected to RNA extraction using the High Pure RNA Paraffin Kit (Roche Diagnostics). cDNA was synthesised from the isolated RNA using the Transcriptor High Fidelity cDNA synthesis kit (Roche Diagnostics). Pre-amplification was carried out using manufacturer's recommendation (Applied Biosystems) with Taqman PreAmp master mix. PCR was carried out using primers as described below. All samples were first quality checked by expression profiling of at least three housekeeping genes (RPL13, GAPDH, β actin) before being used for expression analysis of FRS2 and FRS3 in this study. Statistical comparison was made using the Kruskal Wallis test. *p *< 0.05 was taken as being significant.

### Immunohistochemistry

The tissue microarray (TMA) used in this study has been described [5]. The study cohort included 129 cancers and 36 benign biopsies. All material was used in accordance with approval granted by the local hospital ethical committee. Mouse monoclonal FRS2, (Santa Cruz) and rabbit polyclonal FRS3 antibody (received from Dr S Meakin) used in this study, have been previously validated for use in immunohistochemistry [18,22]. Scoring was done by two independent observers blinded to the clinical detail and the scores collated and analysed by a third investigator (VJG). The score was carried out by assessing the intensity of stain for each core, where immunoreactivity signals was assessed as being absent or weak (0/+) and moderate or strong (++/+++). Data were analysed using the Kruskal-Wallis test. *p *< 0.05 was taken as being statistically significant.

## Results

### Functional redundancy of FRS2 and FRS3 in prostate cancer cells

**Figure 1:**
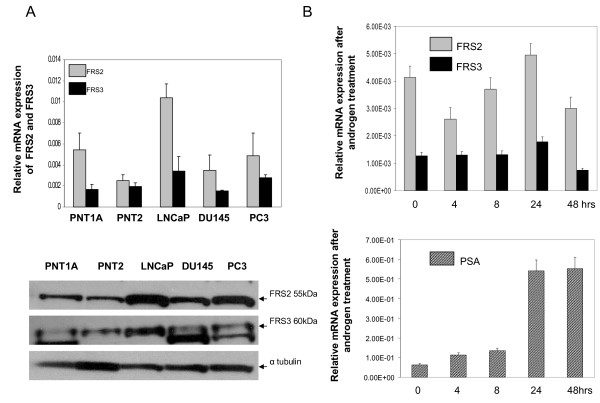
**FRS2 and FRS3 mRNA expression in prostate cell lines**. **A: **FRS2 and FRS3 mRNA expression in benign and malignant cell lines as well as protein expression by western blot using FRS2 and FRS3 specific antibodies. **B: **FRS2 and FRS3 and PSA (positive control) expression in LNCaP cells treated with androgens and assayed at different time points.

**Figure 2:**
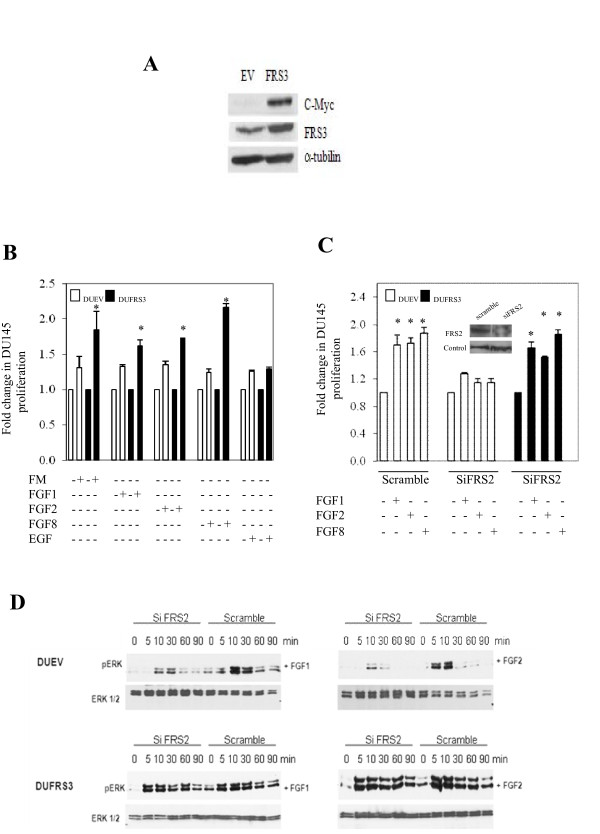
**Mitogenic effect of FRS3 over-expression and functional redundancy**. **A: **Immunoblot analysis showing FRS3-Myc protein over-expression in DU145 cell line compared to empty vector control cell line following stable transfection with FRS3. **B: **FRS3 over-expression enhances cell proliferation in DU145 in response to full media and different FGFs. **C: **FRS2 knock down is compensated for by FRS3 over-expression in proliferation assay in response to different FGF stimulation. Inset image shows immunoblot representative of FRS2 down-regulation following siRNA transfection in DU145 cells. **D: **FRS3 over-expression reverses the inhibitory effect of FRS2 suppression by siRNA on pERK activation. *p < 0.005.

A panel of cell lines was first analysed by real time PCR to define expression levels in prostate cells. FRS2 and FRS3 mRNA were ubiquitously expressed in normal epithelial prostate cell lines (PNT1A/PNT2) and prostate cancer cell lines (LNCaP, DU145 and PC3) (Figure 1A). FRS2 levels were generally higher in all cell lines compared to FRS3 regardless of androgen receptor expression status. A similar pattern of expression in these cell lines was seen in western analysis of FRS2 and FRS3 protein (Figure 1A). We also tested if FRS expression was inducible by androgens. In LNCaP cells neither FRS2 nor FRS3 mRNA expression levels were increased by the addition of androgens suggesting that they are not androgen regulated (Figure 1B). In contrast, simultaneous analysis of PSA expression as a control was significantly increased in LNCaP cells treated with androgens. The function of FRS2 has been well explored in the literature but less is known about FRS3. Given evidence of expression overlap in our cell lines, we tested functional overlap between FRS2 and FRS3 in prostate cancer. A stable myc-tag FRS3 over-expressing clone in DU145 prostate cancer cell lines was first generated (DUFRS3) (Figure 2A). DU145 were selected as an androgen independent cell line with relatively low FRS3 expression compared to FRS2 (Figure 1A). In proliferation assays FRS3 over-expression significantly enhanced cell proliferation in response to stimulation by FGF1 (*p *< 0.005), FGF2 (*p *< 0.001) and FGF8 (*p *< 0.001) compared to empty vector control (Figure 2B). FRS3 has previously been implicated as an inhibitor of EGF signalling [19,20]. In these experiments however increasing FRS3 did not appear to alter (increase or inhibit) the magnitude of EGF stimulated proliferation (Figure 2B). We next asked if FRS3 over-expression could compensate for FRS2 down-regulation as has been previously described in embryogenesis [18]. In serum-starved DUEV cells, the addition of FGFs resulted in enhanced proliferation (*p *< 0.005) (Figure 2C) concomitant with rapid phosphorylation of ERK (Figure 2D, upper panels). Targeted FRS2 knock down reduced this induction regardless of the FGF ligand used (Figure 2C, D upper panels). The effect of silencing FRS2 in the DUFRS3 clone was next tested. Here, over-expression of FRS3 was able to compensate for the effect of targeted knock down of FRS2 when stimulated with different FGFs (Figure 2C). In parallel studies, FRS2 knock down in FRS3 over-expression clones had no discernable effect on ERK phosphorylation (Figure 2D, lower panels). These studies suggest functional redundancy between FRS2 and FRS3 in prostate cancer cells.

### Effect of dual silencing of FRS2 and FRS3

**Figure 3:**
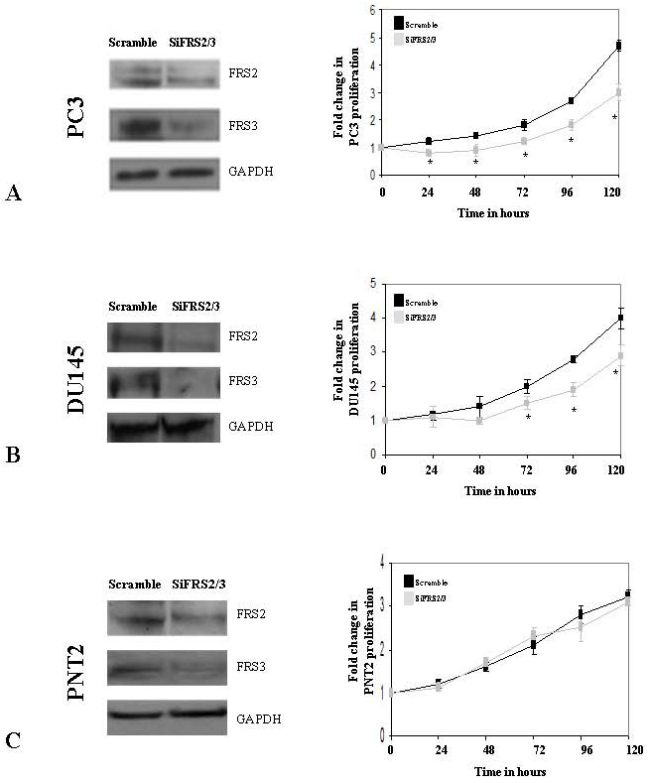
**Effect of dual targeting of FRS2 and FRS3 in cancer and benign cells**. **A: **Immunoblot analysis showing representative dual down-regulation of FRS2 and FRS3 protein in PC3 cell line and corresponding proliferation assay stimulated with full media. **B: **Down-regulation of FRS2 and FRS3 in DU145 cell line and corresponding proliferation assay stimulated with full media. **C**: Down-regulation of FRS2 and FRS3 in benign PNT2 cell line and corresponding proliferation assay stimulated with full media. *p < 0.005. *siFRS2/3- siRNA against FRS2 and FRS3*

**Figure 4:**
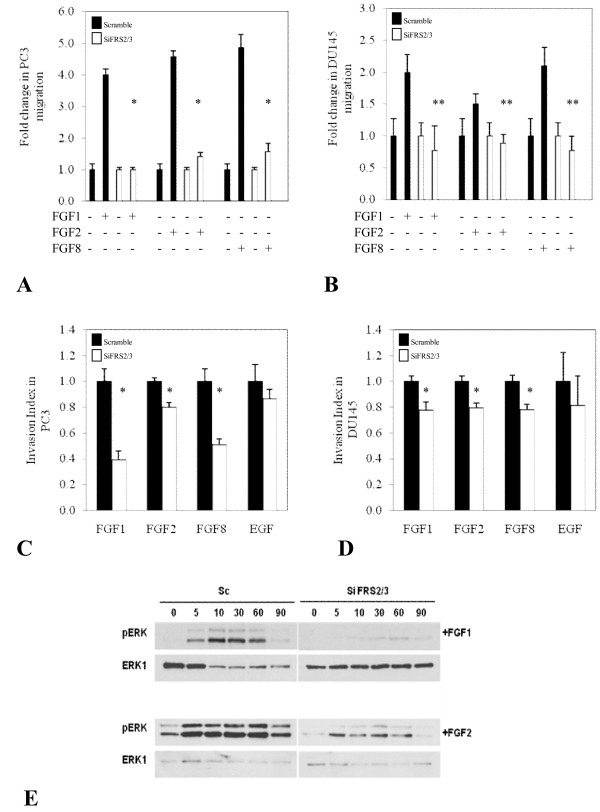
**Dual targeting of FRS2 and FRS3 is a global inhibitor of FGF induction**. **A and B: **Migration experiments in PC3 and DU145 stimulated with different FGFs. **C and D: **Invasion assays for PC3 and DU145 cancer prostate cell lines in the presence of different FGFS and EGF. **E: **Silencing FRS2 and FRS3 inhibits FGF-simulated ERK activation of PC3 cells by FGF1 and FGF2 (*p < 0.005). *siFRS2/3- siRNA against FRS2 and FRS3*

Our results suggest that both FRS2 and FRS3 are important in FGF mediated signalling in prostate cancer. We therefore investigated the effect of dual targeting of FRS2 and FRS3. The efficiency of dual silencing was confirmed at the protein level. Full media stimulation of scramble transfected DU145 and PC3 cells resulted in a significant increase in proliferation compared to un-stimulated cells. Dual silencing of FRS2 and FRS3 in PC3 and DU145 however reduced this induction in comparison to these scramble controls (*p *< 0.005) (Figure 3A and 3B). We did observe that the dual knock down effect was more noticeable in PC3 cells compared to DU145 particularly in the earlier phases of proliferation. This effect could be due to differences in transfection efficiency between the cell lines and/or inherent variability between the cell types in response to FGF stimulation and FRS utilisation. Immunoblot analysis did demonstrate comparable down-regulation of FRS2 and FRS3 protein in both cell lines following transfection. We next repeated the experiments in PNT2 benign prostate cells known to respond to FGF stimulation. In these cells we surprisingly observed that FRS2 and FRS3 suppression did not alter cell proliferation (Figure 3C). We next tested the effect of dual FRS2 and FRS3 silencing in the context of different FGF stimulation in migration and invasion experiments. Treatment of PC3 scramble control cells with FGF1, FGF2 and FGF8 resulted in a significant increase in migration compared to un-stimulated controls. FRS2 and FRS3 dual knock down in these cells however significantly reduced migration regardless of the ligand used in comparison to scramble transfected controls (Figure 4A). Similar results were seen in experiments with DU145 cells (Figure 4B). Invasion assays were next carried out in PC3 cells using FGFs as well as EGF as a chemo-attractant. Dual silencing of FRS2 and FRS3 in PC3 cells reduced the ability of cells to induce invasion upon FGF stimulation (*p *< 0.005) (Figure 4C). No significant change in the invasion index was noted when siRNA FRS2 and FRS3 PC3 cells were stimulated with EGF compared to EGF-stimulated scramble control cells. A similar finding was observed for siRNA FRS2 and FRS3 transfected DU145 cells in invasion studies (Figure 4D) (*p *< 0.05). The effect of dual knock down of FRS2 and FRS3 on FGF induced MAPK activation was next tested. In this experiment, the intensity and duration of ERK phosphorylation following PC3 cell stimulation with either FGF1 or FGF2 in siRNA treated FRS2 and FRS3 cells was dramatically reduced compared to scramble controls in keeping with the functional data observed above (Figure 4E).

### Dual silencing of FRS2 and FRS3 reduces cell recovery after exposure to radiation

**Figure 5:**
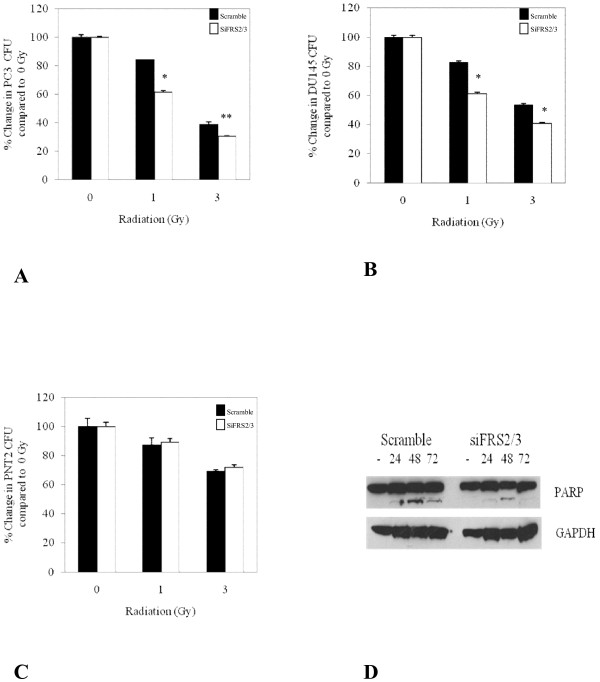
**Dual targeting of FRS2/FRS3 as an adjunct to radiotherapy**. **A and B: **Percentage change in the number of colony forming units in PC3 and DU145 prostate cancer cell lines. **C: **Percentage change in the number of colony forming units in PNT2 prostate cell lines. (* <*0.005*; **p <*0.05*). **D: **Effect of altering FRS2 and FRS3 levels on PARP cleavage in PC3 cells. *siFRS2/3- siRNA against FRS2 and FRS3*

We have observed that combined silencing of FRS2 and FRS3 resulted in a significant inhibition in FGF induced ERK activation in prostate cancer cell lines. MAPK signalling has been shown to play a major role in the cancer cell response and recovery to radiation therapy [23,24]. We therefore tested if targeting FRS2 and FRS3 was a potential therapeutic target in combination with radiation. In these studies silencing FRS2 and FRS3 in PC3 cells significantly inhibited colony formation following irradiation compared to scramble transfected controls (*p *< 0.05) (Figure 5A). A similar finding was observed in siRNA FRS2 and FRS3 transfected DU145 cells (*p *< 0.002) (Figure 5B). These experiments were next repeated with benign PNT2 cells. In contrast to cancer cells, the clonogenic survival after radiation between scramble and siRNA FRS2 and FRS3 cells was not significantly different in these benign epithelial cell lines (Figure 5C). We next tested whether manipulation of FRS2 and FRS3 might have had a direct effect on inducing cell apoptosis. siRNA FRS2 and FRS3 or scramble transfected PC3 cells were assayed by immunoblotting for the extent of PARP cleavage following irradiation (Figure 5D). In this experiment we did not observe any difference in the levels of PARP cleavage in either scramble or siRNA FRS2 and FRS3 transfected samples. These results suggest that FRS2 and FRS3 suppression is not likely to have a direct effect in enhancing apoptosis. Rather, it is more likely that FRS2 and FRS3 suppression may function to inhibit pro-survival signalling which can enhance cell recovery and proliferation in the immediate period after a cytotoxic insult.

### Expression analysis of FRS2 and FRS3 in prostate cancer

**Figure 6:**
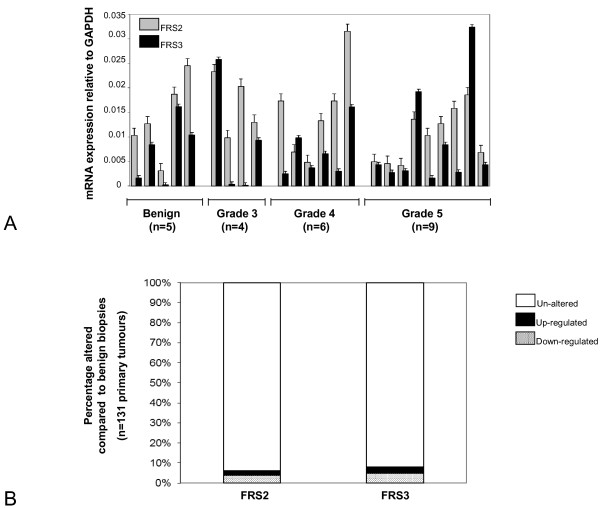
**Expression of FRS2 and FRS3 mRNA in clinical prostate cancer**. **A: **Relative FRS2 and FRS3 mRNA expression in micro-dissected benign and tumours of different Gleason grades. Each profile represents a tumour derived from an individual patient. **B**. Relative alterations in FRS2 and FRS3 expression between benign and malignant prostate samples profiled in the MSKCC Prostate Oncogenome dataset (n = 131 tumours) (26). Here only a small minority of tumours had altered FRS2 and FRS3 mRNA with similar numbers exhibiting up-regulation or down-regulation of each.

**Figure 7:**
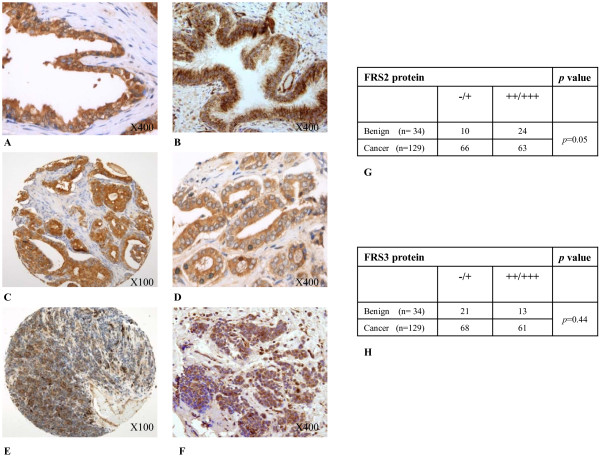
**FRS2 and FRS3 protein expression in clinical prostate tissue**. **A and B: **FRS2 and 3 expression respectively in benign prostate glands **C and D: **FRS2 expression in malignant prostate biopsies. **E and F**: FRS3 expression in malignant prostate biopsies. **G: **Data table on FRS2 protein expression in human prostate tissue stratified by benign or cancer biopsies. **H: **Data table on FRS3 protein expression in human prostate tissue stratified by benign or cancer biopsies.

An important therapeutic question is if either FRS2 or FRS3 are over-expressed in cancer. We therefore tested expression of FRS2 and FRS3 transcript by quantitative real time PCR in a panel of archival clinical prostate cancers using methods previously developed in our group [25]. Benign (n = 5), Grade 3 (n = 4), Grade 4 (n = 6) and Grade 5 (n = 9) tumors each derived from separate individual patients were micro-dissected and RNA extracted and profiled for FRS2 and 3 mRNA expression. In this analysis we found that overall FRS2 transcript levels were higher in benign and malignant biopsies compared to FRS3 consistent with our findings in cell lines (Figure 6A). There was however no difference in expression of FRS2 or FRS3 comparing benign or malignant samples (*p *= 0.8 for both comparisons). We also tested if with increasing cancer grades there was any evidence of a progressive increase or decrease in expression and found no significant alterations (*p *= 0.23 for FRS2 and *p *= 0.87 for FRS3). To further investigate expression in clinical tissue, we interrogated a publicly available dataset. The MSKCC Prostate Oncogenome Project was interrogated for FRS2 and FRS3 transcript expression (n = 131 primary tumours with mRNA data available) using the cBio Cancer Genomics Portal [26]. In this resource (using a Z threshold value of 2.0) there was minimal alteration in FRS2 or FRS3 expression. Only 6% and 8% of tumours had any changes in FRS2 and FRS3 expression compared to normal controls respectively (Figure 6B). These findings corroborate our own data from clinical samples and show that both FRS2 and FRS3 mRNA are expressed in clinical samples suggesting expression redundancy. FRS2 appears to be the predominantly expressed transcript in prostate tissue but neither is significantly over-expressed or down-regulated in the transition to a malignant phenotype. Finally, we next asked if these observations were also true in terms of protein expression. Using a clinical prostate tissue microarray we tested for expression of FRS3 and FRS3 in cohorts of defined cohorts of benign (n = 34) and malignant (n = 129) prostate samples (Figure 7). Comparison of overall expression in these two groups demonstrated no overall significant difference between the groups at the protein level for either FRS2 or FRS3.

## Discussion

Dysregulation of FGF signalling is a common event in many types of cancers. In prostate cancer, FGFR1 and FGFR4 as well as the ligands FGF-1, FGF-2, FGF-6, FGF-8, FGF-9, and FGF-17 are all known to be over-expressed [4,27-31]. The targeting of a common focal point of FGF signalling such as FRS2 and FRS3 to disrupt these diverse components is therefore highly desirable. FRS2 is known to be involved in the activation of MAPK and PI3K signalling, cell proliferation, migration and survival [9,17]. Much less is known about FRS3 and in particular, its function in cancer cells. In prostate cancer cells we observed that FRS3 over-expression was able to induce an enhanced mitogenic phenotype in the presence of FGF stimulation. More importantly, increased FRS3 was able to abrogate the inhibitory effect of forced down-regulation of FRS2. This implies FRS functional redundancy in prostate cancer cells and a potentially important role for FRS3 in maintaining aberrant FGF signalling. Such functional redundancy has not previously been reported in human tumours but is consistent with the findings of Gotoh *et al *whereby weak activation of MAPK by FGF stimulation in FRS2-/- mouse embryonic fibroblast was reversed following ectopic over-expression of FRS3 [18]. FRS2 and FRS3 are attractive targets for manipulation because of their key central role in FGF signalling. An important aim of this study was to test the principle of manipulating FRS. Our results had shown functional redundancy between FRS2 and FRS3 hence siRNA against both FRS were used. Silencing FRS2 and FRS3 had a profound effect on FGF induced proliferation, migration and invasion regardless of the FGF ligand used and in two different cancer cell lines. We did observe some differences in the response of PC3 and DU145 cells to FRS2 and FRS3 suppression. PC3 cells in particular responded better to FGF stimulation and were also more affected by induced FRS down-regulation compared to DU145 cells. Given apparently similar knock-down efficiency from immunoblot assays, it is likely that this difference is due to variability between the cell types in response to FGF stimulation and pathway manipulation. FRS2 and FRS3 suppression in contrast, did not have any appreciable effect on EGF stimulation in either of our prostate cancer cell models. In contrast to this, FRS3 has been reported to inhibit EGF induced-transformation in Saos-2 cells and proliferation in NIH3T3 cells. Suppression has been further reported to enhance the effects of EGF signalling [19,20]. This discrepancy is most likely due to differences in the cell and tumour type studied.

In prostate cancer cell lines and a small cohort of microdissected clinical tumours we failed to find any association between altered FRS2 and FRS3 and the transition to a malignant phenotype. In a larger protein tissue microarray we also did not find any significant association between overall expression and a benign and malignant phenotype. FRS2 and FRS3 therefore do not appear to be altered in prostate cancer. Work in other tumour models have conversely shown differences in FRS2 and FRS3 between benign and cancers with specific down-regulation of FRS3 or FR2 over-expression [19,20,22]. The reason for differences in this pattern is not clear but may represent cell and tumour specific differential utilisation of the FRS proteins. Despite the lack of over-expression we did observe that FRS2 and FRS3 suppression had a significant and specific inhibitory effect in cancer cell lines whereas it had no discernible effect on PNT2 benign prostate cells which are known to express FGF receptors and respond to FGF stimulation [32,33]. Wang *et al *have previously shown that the quantity and quality of FRS2 phosphorylation is dependent on the cellular context and type of activating FGFR [34]. Previous work from our group and that of others have further shown that prostate cancer cells have preferential over-expression of FGFR1 and 4 while FGFR2 and 3 are unaltered or down-regulated [5,27,32,35]. We therefore hypothesise that the observed differential effects from knocking down FRS2 and FRS3 in malignant and benign prostate cells could be due to differences in relative FGFR expression and binding to FRS2 and FRS3 and in turn, corresponding differences in the levels of FRS2 and FRS3 phosphorylation. We acknowledge that PNT2 cells are transformed using a T antigen and may not be a perfect model to test effects in benign cells. Further validation of these observations therefore is needed in non transformed cells such as primary prostate epithelial cells or cells immortalised using a different system (e.g. hTERT) and will be done in our planned future work.

This differential result of FRS manipulation in benign and malignant cells was investigated further as a therapeutic concept in radiation assays. Growth factor induced MAPK activation following radiation exposure is known to have a protective effect from cell death and enhance cell recovery [23,24,36,37]. We tested the principle of manipulating FRS2 and FRS3 as a mechanism of inhibiting MAPK signalling concurrent with radiation therapy. In these experiments we confirmed a synergistic benefit of FRS2 and FRS3 inhibition and radiation therapy. These findings are in agreement with previous studies showing that receptor tyrosine kinase inhibition increases the sensitivity of breast cancer and glioblastoma cells to radiation [38-40]. Crucially, this synergistic effect was only observed in prostate cancer cells and not in benign cells. These results require further validation in planned future work and with stably suppressed FRS2 and FRS3 cells and with different measurements of cell death to test apoptosis. Nevertheless, they do suggest the potential for using FRS2 and FRS3 as a target to achieve selective enhancing of radio-toxicity in tumours with relative sparing of benign tissue. Thus while FRS2 and FRS3 may not be over-expressed in cancer, they may still conceivably be a viable target if their inhibition is selectively effective in cancer but not benign cells.

## Conclusion

In conclusion this is the first study to investigate FRS2 and FRS3 in prostate cancer. FRS2 and FRS3 exhibit functional redundancy in prostate cancer cells and dual inhibition effectively blocks intracellular signalling and the mitogenic effects of multiple FGFs. We also show that neither FRS2 nor FRS3 are apparently over-expressed in cancer and acknowledge that this does limit their attractiveness as a therapeutic target. Preliminary results of down-regulation in combination with a cytotoxic therapy however do suggest that targeting FRS2 and FRS3 may be specifically effective in cancer cells. Taken together we believe that these results justify further studies to investigate FRS2 and FRS3 in FGFR binding and signalling in the prostate and define mechanisms by which FRS2 and FRS3 targeting appears to be more detrimental to cancer cells. These experiments may yet show that they are valid targets to disrupt global FGF signalling most likely as an adjunct to standard cytotoxic therapies.

## Competing interests

The authors declare that they have no competing interests.

## Authors' contributions

VJG conceived, designed and supervised the study and interpreted the data. TV and SD carried out cell based expression studies, experiments and assays, AJ and NK carried out the clinical tumour micro-dissection and expression profiles. SM provided the FRS construct and FRS antibody. VJG and TV drafted the manuscript. All authors have read and approved the manuscript.

## Pre-publication history

The pre-publication history for this paper can be accessed here:

http://www.biomedcentral.com/1471-2407/11/484/prepub
